# Tobacco Use and Periodontal Disease—The Role of Microvascular Dysfunction

**DOI:** 10.3390/biology10050441

**Published:** 2021-05-17

**Authors:** Henrique Silva

**Affiliations:** 1 Informetrics Research Group, Ton Duc Thang University, Ho Chi Minh City 758307, Vietnam; henriquesilva@tdtu.edu.vn; 2 Faculty of Pharmacy, Ton Duc Thang University, Ho Chi Minh City 758307, Vietnam

**Keywords:** periodontal disease, tobacco use, oral microcirculation, nicotine, microvascular morphology, inflammation, angiogenesis

## Abstract

**Simple Summary:**

Periodontal disease consists of a wide range of inflammatory conditions that affect the supporting apparatus of teeth, and is highly prevalent in adults worldwide. Tobacco use is currently recognized as the most important risk factor for periodontal disease as it negatively affects both disease evolution and therapeutic strategies. Given close contact with tobacco products, oral microcirculation becomes dysfunctional which, in turn, aggravates periodontal disease. This paper intends to provide a comprehensive review about the impact of tobacco use on oral microcirculation and the mechanisms underlying periodontal disease aggravation. Acute nicotine administration or tobacco use increases oral perfusion (gingiva, lip, tongue) of healthy subjects due to local irritation and increased blood pressure, which overcome neural- and endocrine-mediated vasoconstriction. Chronic tobacco use, particularly smoking, causes several morphological changes to oral microcirculation, namely, increased vascular density and tortuosity, despite a decrease in capillary diameter, and decreased perfusion due to the multiple vasoconstrictive insults. Periodontal disease involves considerable gingival inflammation and angiogenesis in non-smokers which, in chronic smokers, are considerably suppressed, in part due to local immune suppression and oxidative stress. Tobacco exposure, irrespective of form of use, causes long-term microvascular dysfunction which may not be completely reversible upon cessation, and increases the risk of complications due to the natural disease course or secondary to therapeutic strategies.

**Abstract:**

Periodontal disease consists in highly prevalent wide-ranging inflammatory conditions that affect the supporting apparatus of teeth. Tobacco use is the most important risk factor for periodontal disease as it increases disease severity and periodontal surgery complications. Tobacco use is harmful for the vasculature by causing microvascular dysfunction, which is known to negatively affect periodontal disease. To the author’s knowledge this paper is the first comprehensive review on the mechanisms by which tobacco use affects oral microcirculation and impacts the pathophysiology of periodontal disease. In healthy subjects, acute nicotine administration or tobacco use (smoking/smokeless forms) increases the blood flow in the oral mucosa due to local irritation and increased blood pressure, which overcome neural- and endocrine-mediated vasoconstriction. Chronic tobacco smokers display an increased gingival microvascular density, which is attributed to an increased capillary recruitment, however, these microcirculatory units show higher tortuosity and lower caliber. These morphological changes, together with the repetitive vasoconstrictive insults, contribute to lower gingival perfusion in chronic smokers and do not completely regress upon smoking cessation. In periodontal disease there is considerable gingival inflammation and angiogenesis in non-smokers which, in chronic smokers, are considerably suppressed, in part due to local immune suppression and oxidative stress. Tobacco exposure, irrespective of the form of use, causes long-term microvascular dysfunction that increases the risk of complications due to the natural disease course or secondary therapeutic strategies.

## 1. Introduction

Periodontal disease comprises a wide range of inflammatory conditions that affect the supporting apparatus of teeth (i.e., periodontium), including gingivae, alveolar bone, cementum, and periodontal ligament, which could lead to tooth loss and contribute to systemic inflammation [1]. Periodontal disease is highly prevalent in the adult population worldwide, especially its moderate and mild presentations [2], and has several health implications. For example, it increases the risk of systemic conditions like rheumatoid arthritis and several atherosclerotic cardiovascular diseases [3]. It also increases the risk of mental health, with several studies establishing a link with anxiety and depression [4,5,6,7]. Conversely, it is also known that depression may have a negative outcome in periodontitis patients [8]. The pathophysiology of the disease is chronic and persists with bouts of activity and quiescence to culminate in either the affected tooth falling out or being extracted or the therapeutic removal of dental plaque [9]. Periodontal disease severity depends on several risk factors that are both modifiable and non-modifiable. Non modifiable factors consist mainly in age and genetic susceptibility [10], whereas modifiable ones include poor oral hygiene [11], stress [12], and diseases like diabetes [13,14], although the most recognizable risk factor today is tobacco use [15,16].

Tobacco use, regardless of its form, is associated with a higher risk of developing severer periodontal disease [15,17,18,19]. Several studies have shown that the outcomes of non-surgical and surgical periodontal therapies are compromised in tobacco smokers compared to those who have never smoked [20,21,22,23]. This increased risk affects both active smokers and second-hand (i.e., passive) smokers [24,25]. Former smokers have better periodontal health than active smokers, which suggests that smoking cessation is important for gingival recovery [26,27]. Currently, it is thought that tobacco use increases the risk, pathogenesis and exacerbation of periodontal disease by a combination of several mechanisms: (1) Decreased gingival perfusion, which restricts nutrients and oxygen delivery as well as the removal of waste products; (2) immune response suppression, especially inflammation; (3) suppression of the periodontium’s morphological and functional recovery; and (4) dysbiosis and increased infectivity of oral microbiota. These combined factors impair wound healing and accelerate periodontal disease [26].

Microcirculation consists on the network of blood vessels that are directly responsible for tissue nutrition and waste product removal, besides regulating blood pressure as well as the local immune and hemostatic responses [28]. Tobacco use is known to cause significant microvascular dysfunction in several vascular beds, including oral cavity soft tissues [29,30]. Given the close contact between hazardous tobacco components and oral cavity soft tissues, namely lips, tongue, palate, gingivae, and pharynx, it is well justifiable that tobacco use profoundly affects oral microcirculation, despite the little attention that has been dedicated to this subject.

To the author’s knowledge, this is the first paper to provide a comprehensive up-to-date and critical review on the mechanisms by which tobacco use affects oral microcirculation and impacts the pathophysiology of periodontal disease. Databases on medical science (Pubmed, Springer Link, ScienceDirect, Scopus, and Google Scholar) were searched using combinations of the following keywords: “tobacco”, “smoking”, “nicotine”, “oral”, “lingual”, “gingival”, “microcirculation”, “perfusion”, “periodontal disease”, “periodontitis”. The most relevant research and review papers published between January 1960 and December 2020 were selected and analyzed for this comprehensive review.

## 2. Pathophysiology of Periodontal Disease

Periodontal disease is triggered by the overgrowth of pathogenic bacteria in the oral cavity and their subsequent penetration into local epithelial lining [31]. These bacteria consist of several Gram-negative (e.g., *Porphyromonas gingivalis*, *Treponema denticola*, *Tannerella forsythia*, *Prevotella intermedia and Aggregatibacter actinomycetemcomitans*) and Gram-positive (e.g., *Streptococcus sanguis*, *Streptococcus oralis*, *Streptococcus mutans*, *Actinomyces naeslundii*, and *Actinomyces odontolyticus*). This is followed by appearance of secondary bacteria such as *Fusobacterium nucleatum* [32,33,34]. Several of these bacteria are present in healthy individuals; however shifts of the oral microbiome often associated with poor host health (i.e., poor oral hygiene, tobacco use, etc.) break the symbiotic bond with the host [35]. The subsequent dysbiosis leads to the accumulation of pathogenic bacteria in the subgingival margin and the release of pathogen associated molecular patterns (PAMPs). The resulting local inflammation causes the increase in blood-derived gingival crevicular fluid (GCF), which provides an appropriate environment for the proliferation of bacteria. The subgingival bacterial community growing in the periodontal pocket drives the innate immune response, consisting on the recruitment of neutrophils and natural killer (NK) cells. The resulting cytokine-rich pro-inflammatory environment reinforces the recruitment of more immune cells and the degradation of surrounding tissue [36]. An adaptive immune response ensues, consisting in the uptake of specific antigens by dendritic cells and their presentation to naïve T cells. This results in the formation of T helper cells types 1, 2, and 17, which produce receptor activator of nuclear factor-κB ligand (RANK-L), leading to bone loss [37,38]. Under normal circumstances the pathogenic organisms would be removed, the recruited leucocytes would undergo apoptosis and tissue destruction would be reversible, thus constituting gingivitis. However, under certain conditions that are not fully understood, the pathogenic bacteria continue to replicate and cannot be controlled by the acute immune response, which then becomes chronic and unresolved, resulting in progressive fibrosis and in the irreversible destruction of soft tissue and local bone, thus constituting periodontal disease [39,40].

## 3. Tobacco: Forms of Use and Composition

### 3.1. Forms of Use

Ever since it arrived in Europe in the 15th century, tobacco use has progressively expanded and diversified. Until the 18th century, the most popular forms of tobacco use were smokeless tobacco (i.e., snuff) and pipe smoking. From the 19th century onward, cigarette smoking became the most popular form, and its popularity has grown ever since [41]. However, this increase in popularity also led to several smoking-related diseases, such as chronic pulmonary obstructive disorder [42,43] and cancer, especially lung cancer [44,45]. Smoking is also a risk factor for several other diseases like cardiovascular [46,47] and autoimmune diseases [48,49]. The dramatic number of smoking-related deaths, as well as several anti-smoking campaigns, have led to the development of new forms of nicotine administration that consist in vaporizing a nicotine-containing liquid (electronic cigarettes, e-cigs, and vaporizers, vapers) and in heated tobacco products [41,50]. These new forms of use, marketed as being safer or non-hazardous, have attracted not only former smokers, but also a new generation of consumers [51,52,53]. Despite the growing tendency of these new forms of tobacco use, cigarettes are still the most prevalent form, and are still responsible for a large number of deaths and diseases, professional absenteeism, and a heavy burden for healthcare systems [54,55].

Commercially manufactured cigarettes consist of a tobacco blend with specific ingredients like paper, filter, ink, and adhesive [56]. The blend contains a mixture of different portions of three types of tobacco leaves, namely Virginia/Bright, Burley, and Oriental, which differs in terms of leaf sizes, but most importantly on the curing process itself. Finally, cigarettes also contain additives, which are the substances added to the tobacco blend or ingredients to confer them specific desirable properties and to control cigarette performance while being smoked. Tobacco additives include: humectants to control the moisture level; preservatives to prevent product degradation; fillers; combustion modifiers; and flavorings. Paper additives include modifiers of paper porosity and smoldering rates, as well as hardening agents. When a cigarette is being smoked, two smoke streams form: Mainstream smoke that forms during a puff when air enters the cigarette, and a sidestream smoke that forms between puffs by smoldering from the lit cigarette end. Cigarette smoke is an aerosol of liquid droplets (i.e., the particulate phase) suspended in a mixture of gases and semi-volatile compounds. More than 4700 compounds are found in cigarette smoke, but most are present only in trace amounts [57,58]. Some compounds are found mainly in the particulate phase, such as polycyclic aromatic hydrocarbons, tobacco-specific nitrosamines, phytosterols and metals. Other compounds, termed semi-volatiles, are partitioned between the particulate and gaseous phases. The gaseous phase consists mostly of air constituents, nitrogen and oxygen, with several combustion products like carbon monoxide (CO), carbon dioxide and nitric oxide (NO). There are also other compounds like 1,3-butadiene, formaldehyde, acetaldehyde, acrolein, benzene, and hydrogen cyanide, which are important for their known toxic or carcinogenic properties.

### 3.2. Composition

Unprocessed tobacco leaves contain several secondary alkaloid metabolites, the most prevalent (>95%) of which is nicotine, a secondary alkaloid that acts as an insecticide. It is found largely as levorotary (S)-isomer, whereas only 0.1–0.6% of total nicotine is found as dextrorotary (R)-isomer [59]. Nicotine has a molecular mass of 162.23 g/mol and an octanol/water partition coefficient (logPow) of 1.2 [60]. In commercial cigarette, oral snuff and pipe tobacco nicotine content is about 1.5% per weight, whereas this content is about half in cigars and chewing tobacco [61]. An average tobacco rod contains 10–14 mg of nicotine and, on average, about 1.0–1.5 mg is absorbed systemically during smoking [62]. Since nicotine is a weak base with a pKa of 8.0, nicotine in acidic environments is ionized and does not rapidly cross membranes. This is the case of smoke from flue-cured tobacco that is found in the majority of cigarettes. The pH of that smoke lies between 5.5–6.0, at which nicotine is mostly ionized, and this limits its buccal absorption. Smoke from air-cure tobacco, predominantly used in pipes, cigars and a minority of cigarettes, is more alkaline (pH 6.5 or higher), which facilitates buccal nicotine absorption. Conversely in lung alveoli fluid, pH is about 7.4, at which more nicotine can appear in a neutral form and, therefore, more can be absorbed. The large lung alveoli surface area also increases this absorption. Following lung absorption, nicotine reaches the brain in about 10–20s, where it produces rapid behavioral reinforcement [63,64]. Blood nicotine levels peak when smoking ends [61]. The use of waterpipe tobacco has also grown in the last few decades due to its availability and social acceptability, as well as the possibility of consuming different flavors. However, it is far from safe. A single waterpipe smoking session produces about 46-fold the amount of the tar from a single cigarette [65,66] and leads to five-fold more exhaled carbon dioxide than that of a single cigarette [67]. The blood levels of nicotine of a regular waterpipe smoker are the equivalent to those of people who smoke 10 cigarettes per day [68].

Of the minor alkaloids found in tobacco, nornicotine, and anatabine are the most abundant, followed by anabasine. This order of prevalence appears in cigarettes, oral snuff, chewing, and pipe and cigar tobacco. Nornicotine levels are at their highest in cigar tobacco, anatabine levels are lowest in chewing and oral snuff tobacco, whereas anabasine levels are lowest in chewing tobacco [69]. Other minor alkaloids are thought to arise by bacterial action or oxidation during tobacco processing and not by biosynthetic pathways in the plants [70].

The processing of tobacco leaves to produce cigarettes involves several processes, including drying, milling, mixing, chemical treatment and rolling. This last process consists in adding several compounds to increase nicotine delivery by controlling the burning rate, preserving leaves themselves and modulating organoleptic smoke characteristics. As a result, when a burned commercial cigarette delivers several hundreds of naturally-occurring compounds in leaves and the rolling paper, as well as the additives and compounds that result from burning these compounds [56]. Given this large number of compounds, it is cumbersome to directly attribute negative health effects to one individual compound. Rather the hazardous effects of tobacco smoke result from the combined action of hundreds of these compounds.

## 4. Effects of Nicotine Administration and Tobacco Use on Cardiovascular Function

The cardiovascular responses to the acute and chronic tobacco use are well-established, albeit better characterized for cigarette smoking than other forms of use. Nicotine is thought to evoke the majority of acute physiological responses to tobacco use by acting on cholinergic receptors throughout the organism. By acting on the sympathetic ganglia and adrenal medulla, nicotine evokes the release of noradrenaline and adrenaline [71,72], with plasma levels peaking when smoking ends and then lower [73]. These mediators act on adrenergic receptors, and bring about a rise in both heart rate and peripheral vascular resistance and, hence, of blood pressure [74,75]. This pressor response activates the baroreflex, which conversely inhibits the central sympathetic nervous system [76].

Acute and chronic nicotine administration also produces other endocrine responses such as the stimulation of the secretion of vasopressin, as well as the stimulation of the hypothalamic-pituitary-adrenal and the renin-angiotensin-aldosterone (RAA) axes. These effects, however, are in dependent on the form of administration, as well as on the sex, age and body composition of subjects. The exposure to cigarette smoke increases the levels of vasopressin [77], whereas the isolated administration of nicotine does not [78]. The smoke-induced vasopressin secretion shows a high degree of intersubject variability, probably due to genetic mechanisms [77,78,79,80]. One study found that acute smoking increased vasopressin levels in females, whereas it decreased in males [81]. A similar study reported that smoking-induced vasopressin secretion in healthy subjects was positively enhanced by opioids [82]. The stimulatory effect of smoke on vasopressin secretion also depends on body composition and age. In obese patients, smoke-induced vasopressin secretion was blunted when compared to normal weight subjects and to obese subjects after weight loss [83]. Finally, smoke-induced vasopressin secretion seems to increase with age [84].

Acute administration of isolated nicotine induces the hypothalamic synthesis of corticotropin-releasing hormone [85]. Corticotropin-releasing hormone, vasopressin, and probably also nicotine, bind to specific receptors in the pituitary gland to stimulate the secretion of corticotropin, which increases the secretion of cortisol and corticosterone [86,87]. In addition, corticotropin and vasopressin are also known to evoke the secretion of endothelin-1 (ET-1), a potent vasoconstrictor. In turn, ET-1 further potentiates the release of vasopressin, which reinforces the pressor response of nicotine [74]. The potency of these endocrine responses is probably influenced by the composition of tobacco, namely by the nicotine concentration, as suggested in a recent conducted in young habitual smokers. When smoking a high-tar cigarette (1.6 mg nicotine), the plasma levels of ET-1, corticotropin and cortisol increased significantly after 10, 20, and 30 min, respectively. However, this response was not observed with low-tar cigarettes (0.1 mg nicotine) [74].

Both acute and chronic tobacco smoking are known to activate the RAA axis. In healthy habitual smokers both nicotine inhalation and cigarette smoking (2.2 mg nicotine) increased the activity of the angiotensin-converting enzyme (ACE) and the plasma concentration of aldosterone, whereas renin concentration remained constant [88]. Smoking-induced activation of the RAA axis is supported by a study conducted in human monozygotic twins, which showed higher plasma renin activity and elevated plasma aldosterone concentration in the smoking twin with at least 10 years of continuous cigarette use [89]. There is strong evidence from animal studies to affirm that nicotine administration or exposure to tobacco smoke upregulate ACE, angiotensin II and angiotensin II type 1 receptor (AT_1_R) arm of the RAA axis, which displays pro-hypertensive, pro-inflammatory, profibrotic and sympathostimulatory effects. On the contrary, angiotensin-converting-enzyme 2 (ACE2), angiotensin (1-7) and angiotensin II type 2 receptor (AT_2_R), which display anti-hypertensive, anti-inflammatory, anti-fibrotic and sympathoinhibitory effects are down-regulated [51].

Taken together, these studies highlight the differences between the neuro-endocrine responses to nicotine and to tobacco products, which are observed in the oral microcirculation and will be discussed in the next sections.

## 5. Effects of Nicotine and Tobacco Use on Oral Microcirculation 

Acute effects of tobacco use on oral microcirculation have been assessed in several experimental and clinical studies. Some studies have dealt with the effect of oral mucosa in vivo on microvascular perfusion, whereas others have focused on morphological changes to blood vessels. Given tobacco products’ complex composition, it is difficult to attribute the observed responses to specific components. Even though most studies have suggested that nicotine is responsible for the majority of acute effects on oral perfusion, others have instead attributed these effects to the complex mixture of compounds in tobacco products. From these studies it is clear that effects on perfusion depend in part on the form of tobacco use. Some studies have explored the effects of applying isolated nicotine, snuff or smoking cigarettes, cigars, or even electronic cigarettes, on the microvascular perfusion of oral mucosa by different recording techniques.

### 5.1. Acute Effects of Nicotine on Oral Microvascular Perfusion

The main results of animal studies that have explored acute nicotine effects on oral perfusion in vivo are summarized in Table 1. Whether topically applied or systemically infused via osmotic pumps in dogs for 28 days, nicotine increased perfusion in gingiva [90] and the dental pulp [91]. Similarly, when topically applied to the lingual and cheek mucosa, nicotine increased perfusion at the application site, while lowering at the contralateral site [92]. However, intra-arterial nicotine administration to rabbits decreased gingival perfusion [93,94]. These differences could be attributed to differences in species, nicotine dose, and also to differences in the measurement principles of the different recording techniques.

There are several putative explanations about nicotine effects on oral microvascular perfusion. As nicotine is known to act as a local irritant in several tissues, including oral mucosa [95,96], it has been proposed that it activates sensory neurons to release vasodilator substances, which constitutes the axon reflex [97,98]. In fact, nicotine has been shown to induce the release of calcitonin gene-related peptide (CGRP) from afferent nerve terminals in the rat oral mucosa [99]. Given that CGRP acts as a vasodilator, it is possible that nicotine evokes a transient neurogenic inflammation that increases perfusion. However, this hypothesis does not explain why smokeless tobacco changes perfusion in locations far from the application site [100]. Therefore, it is only logical that neural and/or endocrine responses may also occur. Considering that nicotine induces the release of several vasoconstrictors [71,72], a decrease in perfusion would be expected. However, as oral perfusion actually increases with nicotine, it has been proposed that the increase in blood pressure overrides this vasoconstrictive response [100,101]. 

### 5.2. Acute Effects of Tobacco Use on Oral Microvascular Perfusion

The effects of tobacco on oral microvascular perfusion seem to depend on both the form and duration of use, with most studies having explored the effects of not only cigarette and cigar smoking, but also of vaping and snuff application. For ethical reasons, studies that have assessed the impact of smoked/smokeless tobacco products on oral microcirculation in humans in vivo have employed sporadic-habitual smokers instead of exposing non-smokers to tobacco. Consequently, any comparison between sporadic and habitual smokers is affected by not having a true control group of subjects. To the author’s knowledge only one study has used a sample of non-smoker subjects, and explored the immediate effects of vaping [102]. In most studies conducted in humans, a sham-smoking phase was included before tobacco smoking as the control exposure, and has been determined to assess whether the observed response is attributed to smoke content or to movement-induced (i.e., suction) cardiovascular acute adaptations associated with smoking [98,101,103,104].

The main results of human studies that have explored acute effects of tobacco use on oral perfusion in vivo are summarized in Table 2. Generally, the acute exposure to smokeless tobacco and tobacco smoke resulted in increased gingival perfusion at the assessed site. These results mirror the effects of local nicotine application, even though several other components/factors associated with each type of use can also contribute. When smokeless tobacco (i.e., snuff, 1% nicotine) was applied for 10 min to the gingiva of regular healthy users (mean 25.9 y.o, 1–2 tobacco uses/week), gingival perfusion, quantified as vascular conductance, decreased transitorily during the first minute at the applied site, but then increased significantly throughout the remainder of the application period until 4-minutes post-application [100]. At the contralateral site, a delayed slower increase in perfusion was observed, expressed by the non-significant increase in vascular conductance, and probably affected by the observed wider intersubject variability. As the perfusion increase was noted prior to a rise blood pressure and remained stable after it returned to the baseline values, the authors concluded that true nicotine-mediated vasodilation had occurred and was not mediated by an increase in blood pressure itself. Albeit not hypothesized, axon reflex activation is a possible explanation for the perfusion increase, especially as it is induced in gingiva by several other chemicals [105,106]. At the contralateral site, neural and/or endocrine-mediated vasodilation has been hypothesized. Whether a neural mechanism is present to explain the contralateral increase in perfusion, it is unlikely that it is mediated by the stimulation of beta-adrenergic receptors on gingival blood vessels as previous studies have reported no change in gingival perfusion with propranolol [107]. Another hypothesis is that non adrenergic vasodilator nerve terminals cause this vasodilation [108,109]. Furthermore, a possible crossover of the axon reflex across the midline has also been hypothesized [110]. As no significant increase in vascular conductance occurred, a neural and/or endocrine-mediated response was ruled out and passive pressure-induced hyperemia was reasoned to be the underlying mechanism.

In another study performed with healthy casual smokers (26 y.o., tobacco use on weekends), snuff (1% nicotine) was applied either unilaterally or bilaterally in intact or anesthetized gingiva (mepivacaine, i.e., voltage-gated sodium channel blocker) [111]. When applied unilaterally, snuff increased gingival perfusion at both sites, although it was more pronounced at the application site. When the application was bilateral by keeping one site under anesthesia, bilateral increase also occurred, and was more pronounced at the site without anesthesia, but also at the application site itself during the unilateral application. Similarly, an increase in blood pressure and heart rate was more pronounced during bilateral rather than unilateral application. When applied unilaterally to a superficially anesthetized (lidocaine, i.e., voltage-gated sodium channel blocker) site, gingival perfusion increased bilaterally, and was more pronounced at the application site than at the contralateral site, but was not statistically significant. The authors argued that ipsilateral vasodilation was due to the axon reflex that released vasoactive mediators, whereas contralateral vasodilation was probably of parasympathetic origin. The same study also established that histamine and prostaglandins contributed to the basal blood flow of gingiva because blocking their receptors lowered the baseline perfusion values. However, since neither piroxicam (i.e., non-steroid anti-inflammatory) nor dexchlorpheniramin (i.e., antihistamine) affected vasodilation evoked by snuff, the snuff-induced perfusion increase was not modulated by prostaglandins or histamine. The authors also noted that the subjects who were more used to tobacco presented milder increases in perfusion.

The acute effects of vaping in the lingual microcirculation have been investigated in a pilot study where a small convenience sample of non-smoking subjects (*n* = 10, both genders) was used [102]. Inhaling nicotine-filled vapor (16 mg nicotine/g of e-liquid) significantly increased lingual microvascular perfusion, quantified with laser Doppler flowmetry (LDF), when compared to nicotine-free vapor (e-liquid only). Considering the large intersubject variability reported and the fact that blood pressure was not assessed, no physiological mechanisms for the perfusion increase were proposed by the authors. Nevertheless, it is at least reasonable to assume that an irritation-mediated perfusion increase could have taken place, evoked by nicotine or by e-liquid components [112,113]. 

In young habitual smokers (5–15 cigarettes/day), acute exposure to cigarette smoke exhibited different responses depending on the assessed site. Perfusion was not affected externally at the gingival crest, but increased in the gingival sulcus until the baseline values at 10 min post-smoking. Blood pressure rose during smoking. However, no control group (non-smokers) was employed and considerable intersubject variability was reported [103]. In another study, the authors were able to distinguish between two phases of increased gingival perfusion, once again in healthy casual tobacco users (25 y.o.). During smoking, both perfusion and blood pressure increased, even though vascular conductance decreased. After smoking, blood pressure lowered, whereas perfusion continued to increase. These results suggest that the first response was passive pressure-induced hyperemia that overlapped sympathetically-evoked vasoconstriction, whereas the second response resulted in vasodilation caused by sympathetic response cessation. The observed increased perfusion was bilaterally recorded for most subjects, but perfusion increased only unilaterally in a minority, with different responses at the contralateral site [101]. This suggests considerable anatomical variability between sites in terms of both microvascular architecture and regulation mechanisms. It is well-known that LDF is very sensitive, even to minor changes in vascular architecture, which partly justifies the difficulty in implementation in clinical settings [114]. A later study conducted in middle-aged periodontitis patients, who were smokers and non-smokers alike, found that smoking one cigarette reduced gingival blood flow, which is the opposite result to that found by most previous studies done in subjects with no periodontal disease. This suggests the existence of vascular dysfunction in periodontitis patients regardless of smoking habits [98]. In healthy gingiva of heavy smokers (at least 20 cigarettes/day), light smokers (fewer than 5 cigarettes/day), and non-smokers, no significant differences in gingival perfusion were identified before, during or after smoking, or even in groups [104]. This lack of significance may actually be due to a rise in blood pressure, whose perfusion increase offsets the decrease mediated by sympathetic-mediated vasoconstriction [115].

Finally, it should be considered for all forms of smoked tobacco that the combustion process generates CO, a compound with vasodilator effect, that in part mimics the action of NO, and contributes to lower blood pressure [116,117]. However, considering that acute smoke exposure increases blood pressure, CO may contribute to the acute perfusion increase but not to decrease blood pressure.

The variability between some studies can be attributed to differences in experimental protocols. In one study, an LDF probe with 780–820 nm laser light was used, which penetrates more deeply than 1 mm [104]. However, in several other studies [98,100,101,103,111], a laser Doppler probe with a 633 nm wavelength was employed with a penetration depth between 0.5 and 1 mm. Over a 1 mm depth, contribution from alveolar bone perfusion may come into play, which might hinder gingival perfusion interpretations [118]. Nonetheless, several of these studies mention that perfusion increased in a minority of subjects in the sham smoking phase, which could represent active hyperemia due to the buccal apparatus movements, and possibly owing to recording artifacts [101]. Therefore, this component could also contribute, albeit minimally, to increased perfusion while smoking tobacco.

### 5.3. Chronic Effects of Tobacco Use on Oral Microvascular Perfusion

Current knowledge suggests that acute nicotine exposure creates a transient vasoconstrictor response in oral microcirculation, which is overridden by a concurrent increase in blood pressure. Nevertheless, it is also thought that small, chronic and repetitive vasoconstrictive attacks, as well as revascularization impairment, due to cigarette smoking may contribute to disrupt the immune response and delay healing [101]. In addition, these transient vasoconstrictive phenomena may also lead to a long-term perfusion decrease, limit the delivery of oxygen to tissues and also compromise the ability to remove waste products [119]. Collectively, these chronic changes in oral microcirculation seem to increase the risk of periodontal disease. Several studies have shown that chronic tobacco users, particularly smokers, show a lower bleeding tendency compared to non-smokers, especially in gingiva and the tongue, which has been attributed to a lower perfusion of the oral mucosa. One notable exception is found in a study showing higher perfusion in the Schroeder area of the palate in smokers (10 cigarettes/day) versus non-smokers [120], which might reflect different regulation mechanisms.

The lower gingival bleeding tendency in tobacco users has been attributed to repetitive vasoconstrictive effects of nicotine [121,122,123,124], with several mechanisms being involved. First, the vasoconstrictor effect of nicotine itself needs to be considered. The nicotine-induced release of catecholamines which induce vasoconstriction by acting on alpha adrenergic-1 (α_1_) receptors in vascular smooth muscle [73,125,126,127,128]. Animal models seem to confirm this theory [90,93]. Tobacco smoking is known to elicit constriction in other vascular beds, such as coronary in patients with ischemic cardiac disease [80,129], and in lower limb vasculature [30]. 

Second, decreased perfusion might also be attributed to the reduction in endothelial NO synthesis, which can be due to either the suppression of endothelial nitric oxide synthase (eNOS) expression in the vascular wall by nicotine or cotinine [130], or the decrease in NO mediated by oxidative stress. It is known that the gaseous component of tobacco smoke contains several reactive oxygen species (ROS) generated during combustion. For example, it is estimated that 71–86% of ROS is found in the gaseous form of cigarette smoke, whereas the rest is contained in the particulate form [131]. It is well-accepted that ROS act on the endothelium and increase the production of lipid peroxides which destroy NO and inhibit eNOS to, thus, diminish NO bioavailability [132]. The drop in NO leads to an increased vascular tone, with consequent vasoconstriction and higher blood pressure [133]. Several markers of oxidative stress increase in smokers, and more so in smokers with periodontal disease. Endogenous NO is normally exhaled, which is due to its low-molecular weight and consequent volatility. In current and ex-smokers, however, the NO level in expired air is lower than that of smokers [134]. Conversely, acute smoke exposure increases the level of NO metabolites in exhaled air, namely nitrate [135]. Malonaldehyde is a well-known marker of oxidative stress and its gingival levels rise in periodontal disease patients more than in healthy subjects [136]. Smoking appears to further enhance this oxidative stress by increasing gingival malonaldehyde levels and gingival lipid peroxidation [137]. Malonaldehyde levels in plasma lower after smoking cessation, whereas the levels of several antioxidants, e.g. ascorbic acid, lutein, α-tocopherol, γ-tocopherol, lycopene, and β-carotene, rise [138,139]. When healthy non-smokers smoke a single cigarette, the salivary levels of glutathione (GSH) significantly lower [140]. Similarly, a treatment course for periodontitis significantly decreases glutathione salivary levels in smokers [141]. Thiocyanate salivary levels are higher in periodontitis patients who smoke than in those who do not [142]. Myeloperoxidase activity in GCF, which is considered a marker of periodontitis, is also higher in smokers [143,144]. This oxidative stress is thought to be a reason for worsening periodontal disease in smokers as the levels of several antioxidant compounds alter in smokers [145]. Therefore, the depletion of important antioxidants may facilitate the ROS-mediated depletion of endothelial NO. In fact, the link between smoking and oxidative stress in the oral mucosa of patients with periodontal disease has already been established [145].

However, there may be other factors that explain lower perfusion in smokers, including local mediators such as angiotensin II, ET-1, histamine, and prostaglandins. Despite the presence of receptors for ET-1 and angiotensin II in the periodontium, there is currently no knowledge that these mediators contribute to the regulation of gingival basal vascular tone or to tobacco use-mediated perfusion changes [146]. One study showed that histamine and prostaglandins contribute to tonic gingival perfusion [100], whereby a decrease in these mediators can result in the loss of vascular homeostasis. In fact, chronic smokers present lower plasma prostacyclin levels than non-smokers [147]. Histamine is known to act on endothelial cells and stimulate the release of prostacyclin and endothelium-derived hyperpolarization factors (EDHFs) [148]. In the oral cavity, histamine is produced by gingival fibroblasts, neutrophils and macrophages, and its secretion increases when exposed to bacterial and viral products [149]. Even though salivary histamine has not yet been established as a reliable periodontitis marker, it is high in periodontitis patients [150], and smoking further contributes to increase it [151]. In healthy regular smokers, gingival blood significantly increases 3 days after quitting, with further increases over the following weeks (8 weeks in the study) [152]. This suggests that the effect of long-term smoking is, in fact, decreased gingival perfusion.

Besides lowering resting perfusion, chronic tobacco use also changes local microvascular reactivity to several stimuli when applied to oral mucosa, including vasoconstrictor drugs [153], and inflammatory stimuli like heating [154] and dental plaque accumulation [155,156]. Gingival/periodontal inflammation can be assessed by diverse techniques and parameters, such as quantifying gingival bleeding upon probing (BOP) and GCF. The BOP parameter is known to be low in smokers [157,158], probably by the long-term perfusion decrease that hinders inflammation. Gingival crevicular fluid is an extracellular fluid that accumulates between gingiva and tooth cementum. Its secretion depends on the local Starling forces in gingival microcirculation, and accumulates with vasodilation, which accompanies inflammation [159]. One study showed that GCF production evoked by heat-induced gingival inflammation correlated well with several perfusion-dependent parameters in non-smoking subjects, whereas no such correlation was possible in smokers, probably due to the inflammation-impairment effect of smoking [154]. Nevertheless, the composition of GCF seems to be affected by tobacco use, with cigarette smokers showing higher levels of pro-inflammatory cytokines than electronic cigarette smokers and never smokers [160]. Bacterial plaque accumulation evokes local gingival inflammation, the so-called plaque-induced gingivitis, which consequently increases perfusion. In smokers, this plaque-induced vasodilation is suppressed to half its intensity [155]. Gingival microvascular reactivity to vasoconstrictor drugs is altered is also smokers. One study has demonstrated that smokers seem to respond differently to a local anesthetic containing lidocaine and adrenaline (i.e., a known vasoconstrictor), than non-smokers. This suggests that although no clinical manifestations are present, smokers’ gingiva may already show signs of microvascular dysfunction [153]. However, smokers display lower perfusion in gingivae and the tongue, as several studies observed that were conducted by LDF [161]. Although LDF has been shown to be adequate for perfusion assessments in transversal studies in periodontics, its usefulness for long-term assessments has been debated recently. A study has reported that LDF displays acceptable reproducibility for assessing gingival perfusion [162]. However, it was conducted in a small convenience sample of non-randomly chosen middle-aged non-smoker subjects (*n* = 10, mean age 50 y.o., both genders). Considering its limitations, it cannot be assumed that an equally acceptable reproducibility will be achieved in studies involving smokers, especially when the many known factors that determine the variability of the LDF signal still need to be evaluated, such as the spatial variability, the influence of sex hormones on microvascular reactivity of oral mucosa, to name a few [163].

Several sources of variability need to be considered when examining the effects of smoking. First, some discrepancy appears when classifying subjects as “heavy” or “light” smokers. In some studies, this classification is based on the number of cigarettes smoked per day [98,101,103], whereas others base it on the plasma/urine/salivary levels of cotinine [104,126]. By considering inter-individual variability in terms of the hepatic cytochrome P450 (CYP) metabolism of nicotine [164], certain subjects could have been wrongly classified. Second, most studies do not mention of the time that oral hygiene practices lasted before measuring oral blood flow. It has been shown that mechanical stimulation with a toothbrush increases gingival oxygen tension [165], gingival blood flow, and GCF [166]. Considering that these practices can cause significant trauma to gingiva at least, even without presenting any considerable clinical changes, this is another factor to include in on this discussion.

### 5.4. Effects of Tobacco Use on Oral Microvascular Morphology

The effects of tobacco use, especially smoking, on the microvascular morphology have been consistently described in several organs. Tobacco smoke has been reported to cause the thickening of arterioles in the trachea, lung, esophagus, stomach, myocardium, pancreas, and kidney [167,168]. Furthermore, vasoconstriction and edema secondary to endothelial dysfunction have also been described in the placenta [169,170,171] and intervertebral disks [172].

Different studies have reported several morphological changes in oral microcirculation, namely in gingival, lingual and labial beds [173,174,175,176,177,178,179], which are presented in Table 3. These studies have been performed mainly by histomorphometric analysis, videocapillaroscopy (VC), stereomicroscopy, and orthogonal polarization spectral imaging (OPSI) techniques, and quantify capillary density, vessel caliber and tortuosity level. In the gingival microcirculation of young smokers, no changes in gingival capillary density have been found between smokers and age-matched non-smokers. In one study seven young female smokers (25–38 y.o.) with a mean tobacco history of ~13 years (~16 cigarettes/day) did not show significant differences in terms of capillary density when compared to age- and gender-matched non-smokers [180]. In a more recent study employing 10 young male subjects (mean age 25 y.o.) with a history of 15–25 cigarettes a day for the last 5 years, again no changes in gingival capillary density have been found with age- and gender-matched non-smokers, as assessed by OPSI [173]. This suggests that in young subjects microcirculation is still morphologically intact and does not show readily observable lesions.

Studies conducted in older subjects, however, have shown important differences in the microvascular architecture between smokers and non-smokers, however depending on the employed technique. Using VC as a quantification technique a study reported significantly higher capillary density in the gingival mucosa of chronic middle-aged smokers when compared to non-smokers, together with smaller and more tortuous capillaries [181]. Furthermore, another study reported that these morphological changes persisted in the microcirculation of ex-smokers (mean smoking duration of 17.28 years) even after an average 13-year smoking cessation period [182]. The same technique showed capillaries with a smaller caliber, but a higher density and tortuosity in the lingual microcirculation of chronic cigar smokers (age 56–72 y.o.) [176] and in the labial mucosa of middle-aged cigarette smokers (mean age 43 y.o.) [174]. However, two studies using histomorphometric analysis failed to show significant differences in the morphology of gingival microcirculation in samples with similar sizes and composed of smokers with comparable ages [178,179]. These studies suggest that VC is more reliable than histomorphometric analysis for the identification of the morphological changes in the oral microcirculation that occur with chronic smoking. Nevertheless, differences in the anatomical site for sample collection may also explain these differences in sensitivity. Finally, these morphological changes may not be completely reversible with smoking cessation, which should be clarified with studies employing subjects with different smoking durations and even longer cessation periods. 

Several mechanisms seem to be at play to explain these morphological changes in oral microcirculation. The increased capillary thickening and accompanying tortuosity can be attributed to an increased vascular mitogenesis. The systemic administration of nicotine, either short-term (24 h) or long-term (2 weeks), is known to decrease both the length and height of the capillary fragments examined histologically [183]. Additionally, both nicotine and cotinine up-regulate the vascular endothelial growth factor (VEGF) at mRNA and protein levels in endothelial cells [184,185]. They have a minor mitogenic effect on vascular smooth-muscle cells [186], where they potentiate the secretion of basic fibroblast growth factor (b-FGF) and matrix metalloproteinases, which are critical for cell migration [187]. These effects could justify the increase in vascular thickness in the oral tissues of regular tobacco users free of periodontal disease.

The increased capillary density seems to be attributed to the recruitment of underperfused capillaries, probably due to a combination of low oxygen tension and increased post-capillary venous pressure. It is well-known that tobacco smoking delivers low CO levels to the blood which results in a dose-dependent decrease in oxyhemoglobin and an increase in carboxyhemoglobin. Although oxyhemoglobin levels lower only slightly, CO also enhances the hemoglobin-oxygen binding affinity, which results in lower oxygen partial pressure [188], to which the repetitive vasoconstrictive episodes during smoking probably also contribute. Tissue hypoxia has been firmly established to evoke a compensatory increase in the functional capillary density [189]. In addition, chronic exposure to tobacco smoke has been shown to increase postcapillary venous pressure but not precapillary arterial pressure in the rat mesenteric microcirculation [190]. This increase in venous pressure can in turn lead to the recruitment of underperfused capillaries [174], similarly to what occurs in peripheral venous insufficiency and critical limb ischemia [191,192]. Given that regular smokers show lower gingival perfusion, less oxygen hemoglobin saturation and lower oxygen content of periodontal pockets when compared to non-smokers [161,193], it is only logical to assume that capillary recruitment should explain the observed density increase in long-term exposure to tobacco smoke. Still, despite the increased density, these capillaries display reduced diameters, which should justify the overall perfusion decrease in oral microcirculation in chronic smokers.

### 5.5. Effects of Tobacco Use on the Vascular Endothelial Adhesive Properties

Tobacco components are known to have significant toxic effects on endothelial cells in vitro by inducing oxidative stress by ROS [194], and even causing necrosis [195]. A reflection of this oxidative stress-mediated injury is increased superoxide radical production in human umbilical vein endothelial cells (HUVECs) from smokers versus those from non-smokers [196]. Treatment of HUVECs with plasma exposed to cigarette smoke leads to oxidative injury, which results in GSH and ACE extending to the medium, and in a smaller cellular ATP pool [197]. In addition to oxidative stress, tobacco extracts inhibit the viability HUVECs in a dose-dependent manner, and induce injury by promoting cytokine release, DNA damage and apoptosis [198,199]. Recently, major toxic effects in HUVECs have been identified in the compounds responsible for aroma electronic cigarettes [200].

It is known that tobacco use also changes the adhesive profile of endothelial cells by increasing the expression of the surface proteins that promote the attraction of circulating leucocytes to, thus, facilitate the initiation or maintenance of vascular inflammation. Exposing HUVECs to cigarette smoke condensate or extract increases the expression of intercellular adhesion molecule-1 (ICAM-1), vascular cell adhesion protein 1 (VCAM-1) and E-selectin by the mitogen-associated protein kinase (MAPK)-independent pathway [201,202], while down-regulating the expression of anti-inflammatory cytokines like growth-related oncogene, interleukin (IL)-6, and monocyte chemoattractant protein-1 (MCP-1)[203]. When exposing HUVECs to smokeless tobacco extracts, the expression of E-selectin, interleukin-8 and of MCP-1 increases, and neutrophils migrate avidly across these cells compared to those not exposed [204]. This change in endothelial cell phenotype may also result from indirect action mediated by vascular macrophages. In fact macrophages exposed to cigarette smog express higher tumor necrosis factor-alpha (TNF-α) levels which, in turn, also contributes to increase ICAM-1 expression in endothelial cells [205]. 

The results of periodontal tissue samples obtained from smokers further support these findings in HUVECs. Intercellular adhesion molecule-1 is normally expressed on the endothelial cell surface of gingival blood vessels, and plays an important role in controlling the trafficking of leukocytes to gingival tissue. Acute cigarette smoke exposure does not seem to change ICAM-1 serum levels despite the existing correlation between serum ICAM-1 and serum cotinine levels [126]. It has been found that serum ICAM-1 levels significantly rose in regular smokers versus age-matched non-smoking subjects [126,206,207]. Conversely, lower ICAM-1 levels have been detected in the GCF of smoking periodontitis patients compared to non-smoking patients [208] and also in smokers’ healthy periodontal tissue [175]. However, inflamed periodontal vessels express higher ICAM-1 and E-selectin than healthy vasculature, with no differences between smokers and non-smokers [175]. These results suggest that inflammation is the main factor responsible for increasing the ICAM-1 expression in the gingival vasculature, irrespectively of smoking status. In addition, low basal periodontal ICAM-1 expression may reflect the shedding of membrane-bound protein, although it may also result from adapting to nicotine exposure [209]. 

There are reports of significant exposure to tobacco products causing such a degree of vascular endothelial cell lesion that it causes the detachment of endothelial cells into circulation [210,211]. For example, electronic cigarettes have been recently associated with a bigger number of endothelial cells in the bloodstream [212], probably due to the cytotoxic effect of certain components in these devices. Interestingly, heated tobacco products do not appear to have any measurable effects on HUVECs’ viability or migration ability [213]. This endothelial detachment generates areas of the exposed subendothelial matrix, which attracts platelets. They secrete platelet-derived growth factor (PDGF), a mitogen that leads to vascular smooth-muscle cell hyperplasia. Although endothelial cell detachment increases the risk of platelet adhesion and possible thrombotic events, no such link has yet been established. Cigarette smoke condensate induces endothelial cells to secrete von Willebrand factor in a time-dependent way [198], which further increases the risk of thrombosis. This endothelial lesion triggers repair mechanisms mediated by endothelial progenitor cells. Regular cigarette smokers have a few endothelial progenitor cells in serum, together with faulty differentiation and functional impairment, which shows significant impairment [214]. Although electronic cigarettes are perceived as “safe” by the general public, it is known that even one puff increases the level of endothelial progenitor cells in blood [215].

Blood rheology is affected by tobacco smoking [216,217] which, in turn, favors the expression of VCAM-1 and MCP-1, which also increases leucocyte attraction [218]. This rheology change also leads to higher vascular shear stress, which activates the classic complement pathway [219]. Tobacco smoke is also known to activate the complement pathway, particularly the alternative pathway in vitro [220]. In fact tobacco smoke promotes the deposition of complement component C4 on the surface of human endothelial cells [221].

### 5.6. Chronic Effects of Tobacco Use on Periodontal Inflammation

In patients with periodontal disease there is a marked increase in gingival perfusion, which has been attributed to the combination of a chronic inflammatory reaction coupled with stimulated angiogenesis. In periodontal disease there is considerable infiltration of leukocytes in the gingival interstitium with the release of pro-inflammatory cytokines and chemokines. Activated neutrophils, macrophages and lymphocytes, as well as gingival endothelial cells overexpress the inducible form of NO synthase (iNOS), with the large amounts of NO released contributing to vasodilation as well as to periodontium destruction [222].

The injury to the gingival keratinocytes and endothelial cells increases the expression of ET-1, which also increases in GCF [223] and is itself responsible for inducing the expression of several pro-inflammatory cytokines (e.g., interleukins 1β and 6, and tumor necrosis factor-alpha), thereby maintaining the inflammatory status [224]. This increased ET-1 expression can also be attributed to the decreased expression of ET-1 inhibiting mediators. For example, the pro-angiogenic factor angiopoietin-1, a known inhibitor of ET-1, is found in lower levels in subjects affected with a more severe form of periodontal disease [225,226]. Finally, a frequently present bacterial species, *Porphyromonas gingivalis*, expresses PgPepO, an endopeptidase with significant homology with endothelin-converting enzyme, which converts the endothelin precursors into their active forms [227]. Thus, this species may help explain the increased endothelin load in periodontal disease. Furthermore, there is also a neurogenic component that contributes to the inflammatory process, with the concomitant release of neuropeptides such as substance P (SP), CGRP, and vasoactive intestinal peptide (VIP), which also contribute to vasodilation. Vasoactive intestinal peptide and SP accumulate in the gingival tissue and their levels in the GCF increase throughout the course of periodontal disease [228]. Calcitonin gene-related peptide is degraded in the GCF, which causes its levels to decrease [229].

Chronic exposure to tobacco, particularly smoking, enhances dysbiosis and leads to a suppression of the immune response, thus contributing to an enhanced susceptibility to periodontal disease. Smokers exhibit a decrease in several pro-inflammatory cytokines and chemokines and certain regulators of T-cells and NK-cells [230]. Smokers appear to have depressed numbers of T-helper lymphocytes [231], important to B-cell function and antibody production, as well in mast cells [232]. Smoking seems to differently affect neutrophil function, generally preventing pathogen removal from periodontal pockets. However, in heavy smokers the high amount of generated ROS and consequent oxidative stress contribute to tissue damage [233].

The effects of smoking on oral microbiome are somewhat controversial, with some studies showing important differences in the microbiome of smokers and non-smokers, whereas others fail to show any significant differences. This variability has been attributed to differences in study design, especially regarding the sensitivity and specificity of the microbiological methods employed. Nevertheless, it is clear that smoking exposure creates a stressful environment to which periodontal pathogens, notably *Porphyromonas gingivalis* can adapt by changing their gene and protein expressions. This, in turn, may alter the virulence of bacteria and host-pathogen interactions, promoting a pathogen-enriched microflora in periodontal disease patients which is more resistant to treatment. The mechanisms underlying this smoking-induced dysbiosis are, unfortunately, not understood and still open for discussion [234].

### 5.7. Chronic Effects of Tobacco Use on Periodontal Angiogenesis

Besides an increased expression of vasodilators, periodontal disease is also characterized by potentiation of angiogenesis, which is translated by the increased levels of several pro-angiogenic mediators. The salivary levels and gingival expression of angiogenesis-promoting mediators such as vascular endothelial growth factor (VEGF) and basic fibroblast growth factor (b-FGF) were found to be elevated in patients with periodontal disease [235,236,237]. Vascular endothelial growth factor levels are increased in plasma [238], saliva [237], GCF [239,240,241], and in the gingival epithelial and stromal compartments [235,236,242], and correlate with disease progression and severity. Basic fibroblast growth factor is a pro-angiogenic mediator also involved in tissue regeneration and its levels are increased is the saliva [237] and GCF [243] of patients with periodontal disease. This potentiation of angiogenesis increases capillary density [244] and justifies in part the increased bleeding tendency.

Long-term tobacco use, particularly smoking, has been repetitively associated with suppression of the angiogenesis process, both in healthy subjects as well as in periodontal disease patients. This in part justifies the lower bleeding tendency in smokers, even without periodontal disease and with apparently healthy gingiva [245,246]. This suppression of angiogenesis is supported by observation of significant changes in the levels of pro-angiogenic mediators between smokers and non-smokers, notably VEGF and b-FGF. In healthy subjects, salivary levels and gingival expression of b-FGF and VEGF are significantly lower in smokers than in non-smokers [232,237]. An in vitro study in endothelial progenitor cells has shown that the ROS generated by tobacco smoking contribute to the suppression of the Akt/eNOS/NO pathway and to the decreased expression of integrins and of VEGF [214]. This in turn contributes to the decreased ability of endothelial cell migration and tube formation, essential steps of the angiogenesis process. Additionally, in alveolar macrophages from long-term smokers it has been shown that the expression of VEGF is significantly lower when compared to age-matched non-smokers [247]. These in vitro results once again stress the differences between the effects of isolated nicotine/cotinine and the global effects of the many components of smoke. Even though nicotine/cotinine are able to upregulate VEGF in endothelial cells [184,185], the ROS produced during smoking are enough to offset these effects and to overall depress VEGF expression. In vivo studies have shown contradictory results with regards to the impact of tobacco use on VEGF levels of healthy subjects. In a study evaluating smokers of both genders (*n* = 82, mean age 53 y.o.) smoking at least five cigarettes a day for more than 6 months, no significant differences in plasma VEGF were detected when compared with age-matched non-smokers [248]. Similarly, when comparing smokers of both genders (*n* = 22, mean age 38 y.o.) with a six pack-year history, smoking at least 10 cigarettes/day during the previous year, again no significant differences in plasma VEGF levels were found. However, there was a significant inverse correlation between VEGF levels and endothelium-dependent vasodilation, suggesting nevertheless the relevance of VEGF levels for vascular functional status [249]. However, in a group of adolescents (*n* = 310, mean age 14 y.o.) that regularly smoked cigarettes or waterpipe tobacco significantly lower plasma levels of VEGF were found in boys but not in girls when compared with non-smokers [250]. These differences in terms of VEGF values may be partly justified by the differences in terms of study design, suggesting that subjects’ age and gender, as well as type and longevity of tobacco use may be important factors to consider when studying and should be better controlled in future studies.

Tobacco use also suppresses angiogenesis an inflammation in periodontal disease patients [251,252]. This seems to explain their reduced bleeding tendency and, consequently, the wound healing impairment and the acceleration of the disease itself [26]. In periodontitis patients, smokers show lower gingival perfusion than that non-smokers [253]. Consistently with this, gum bleeding upon gentle probing is lower in smokers [27,125,245,254] and increases toward non-smoker levels after smoking cessation [255]. Gingival probing shows less bleeding in smokers than in non-smokers with the same amount of dental plaque [251]. Another study has shown a weaker correlation between the visible plaque index and the gingival bleeding index in smokers than in never smokers [256]. The gingival probe penetration depth is less in smokers than in non-smokers, probably due to fibrosis [257]. Smoking cessation increases not only gingival perfusion and bleeding upon probing after a few weeks, but also the crevicular volume and flow rate [255]. These clinical observations are again supported by significant differences in the levels of angiogenic mediators between smokers and non-smokers. Plasma VEGF levels have been shown to be higher in periodontal disease patients who are non-smokers when compared to smokers [258]. Furthermore, salivary endoglin, ICAM-1, and platelet endothelial cell adhesion molecule-1 (PECAM-1) levels as well as gingival VEGF expression are reduced in patients who are smokers in comparison to non-smokers [232,237]. Therefore, the impact of tobacco use appears to promote angiogenesis in periodontal disease patients who are non-smokers and to suppress the process in patients who are smokers.

## 6. Conclusions

Tobacco use is recognized as the most relevant risk factor for periodontal disease. Exposure to nicotine or to tobacco products evoke different responses in oral microcirculation, highlighting the importance of many substances besides nicotine. In healthy subjects, acute exposure to nicotine or tobacco products increases gingival and lingual perfusion due to a combination of local irritation and blood pressure increase, which override nicotine-induced vasoconstriction. Chronic tobacco use decreases perfusion due to repetitive vasoconstrictive insults and to a remodeling effect in microvasculature. In periodontal disease, microbe-mediated tissue destruction induces overexpression of endothelial adhesion molecules which increase leucocyte attraction to create chronic inflammation and stimulate angiogenesis. These processes are suppressed in patients who are chronic tobacco users, due to the decreased expression of pro-inflammatory cytokines and pro-angiogenic factors, probably attributed to oxidative stress. This justifies the reduced bleeding tendency and the increased risk of complications in patients who are smokers. Regardless of the form by which tobacco is used, it causes long-term functional and morphological changes to oral microcirculation, which may not completely reverse upon cessation. 

## Figures and Tables

**Table 1:** Description of the main results of the most relevant studies into the effect of nicotine on oral microcirculatory perfusion in vivo.

Authors	Species/Strain	Nicotine Dose and Administration Route	Measurement Site	Assessment Technique	Main Results
Clarke et al. (1981) [93]	New-Zealand lop-eared rabbits under urethane anesthesia	Intra-arterial administration (right common carotid artery)	Gingiva	Thermal-diffusion transducer	Perfusion decrease
Clarke et al. (1984) [94]	New-Zealand lop-eared rabbits under urethane anesthesia	Systemic administration (16.2 μg/mL) via infusion pump. Ten infusions were given at 30 min intervals over a 5-hour period. Infusions were repeated over a 6-month period	Gingiva	Thermal-diffusion transducer	Perfusion decrease
Huckabee et al. (1993) [92]	Dogs under sodium thiamylal anesthesia	Topical administration of moist snuff containing 3.12, 6.25, 12.5, 25, 50, and 100 mg/kg of nicotine for 7 min	Cheek mucosa and tongue	Radiolabeled microsphere method	Perfusion increase at the application site and decrease at the contralateral site.
Johnson et al. (1991) [90]	Dogs	Topical (8 mg/kg/day) administration for 28 days	Mandibular gingiva	Radiolabeled microsphere method	Perfusion increase in anterior mandibular gingiva regardless of the administration route
		Systemic (2.5 mg/kg/day) administration by subcutaneous osmotic mini-pumps			
Johnson et al. (1993) [91]	Dogs	Topical (8 mg/kg/day) administration for 28 days	Anterior mandibular gingiva	Radiolabeled microsphere method	Perfusion increase regardless of the administration route
		Systemic (2.5 mg/kg/day) administration by subcutaneous osmotic mini-pumps for 28 days			

**Table 2:** Description of the main results of the most relevant studies into the acute effects of tobacco products on oral microcirculatory perfusion in vivo (y.o.—years old; SBP—systolic blood pressure; DBP—diastolic blood pressure).

Authors	Subjects (Sample Size; Mean Age; Tobacco Habits)	Tobacco Product	Assessment Site	Assessment Technique	Main Results
Baab et al. (1987) [103]	Healthy habitual smokers (*n* = 12, 22.4 y.o., 5–15/day for 2–8 years)	Cigarette	Gingival margin and forearm skin	Laser Doppler flowmetry	Increased gingival blood flow, SBP and DBP—blood flow returned to the baseline after 10 min. Reduced forearm blood flow.
Meekin et al. (2000) [104]	Healthy habitual smokers (*n* = 15, mean age 34–36 y.o., 6 light smokers, 9 heavy smokers)	Filterless cigarette	Gingival and forehead skin	Laser Doppler flowmetry	Significant increase in forehead perfusion in light smokers. Non-significant perfusion increase in gingiva in all the groups
Mavropoulos et al. (2001) [100]	Healthy habitual tobacco consumers (*n* = 22, 25.9 y.o.)	Smokeless tobacco (snuff)	Gingiva, applied unilaterally	Laser Doppler flowmetry	Blood flow increase at the applied and contralateral sites. Heart rate and blood pressure increased. Neural or endocrine mechanism may be involved.
Mavropoulos et al. (2002) [111]	Healthy human subjects (*n* = 18, 26 y.o.)	500 mg of snuff (1% nicotine)	Buccal maxillary gingiva; skin of the forehead and thumb	Laser Doppler flowmetry	Rapid increase in gingival and blood flow. Blood pressure and heart rate increased. Vasodilation was attenuated by infraorbital nerve block (mepivacaine)
Mavropoulos et al. (2003) [101]	Humans, healthy casual smokers (*n* = 13)	Cigarette smoke	Gingiva and thumb and forehead skin	Laser Doppler flowmetry	Vasoconstriction in gingiva, overcome by increased blood pressure, which led to a higher blood flow.

**Table 3:** Description of the main results of the most relevant studies into the effect of tobacco products on the oral microvascular morphology in vivo (y.o.—years old).

Authors	Subjects (Sample Size; Mean Age; Tobacco Habits)	Assessment Site	Assessment Technique	Main Results
Persson et al. (1988) [180]	Healthy habitual female smokers (*n* = 7, 33.6 y.o., mean 16.1/day for a mean of 13.1 years)	Gingival margin of the mandibular and maxillary anterior regions	Stereophotography	No significant differences in capillary density when compared to age-matched non-smokers
Lindeboom et al. (2005) [173]	Healthy habitual male smokers (*n* = 10, 25.0 y.o., 15–25/day in the previous 5 years)	Gingival margin (buccal aspect) of the first right maxillary premolar region	Orthogonal polarization spectral imaging	No significant differences in capillary density when compared to age- and gender-matched non-smokers
Scardina et al. (2019) [182]	Healthy ex-smokers (*n* = 25, 58.4 y.o., smoking duration of 17.28 years, cessation duration of 13.28 years)	Gingival mucosa	Videocapillaroscopy	Significantly higher capillary density, smaller and more tortuous capillaries in ex-smokers and in smokers when compared to age-matched non-smokers
Scardina et al. (2005) [176]	Healthy cigar smokers (*n* = 25, 56.7 y.o.	Lingual mucosa	Videocapillaroscopy	Significantly higher capillary density and tortuosity and lower caliber when compared with age-matched non-smokers
Lova et al. (2002) [174]	Healthy cigarette smokers	Labial mucosa	Videocapillaroscopy	Significantly higher capillary density and tortuosity and lower caliber when compared with age-matched non-smokers
Sönmez et al. (2003) [178]	Cigarette smokers with periodontitis (*n* = 38, 38 y.o., from less than 10 to more than 20 years of smoking)	Gingival mucosa	Histomorphometric analysis	No significant changes in vascular density when compared with age-matched non-smokers
Kumar et al. (2011) [179]	Cigarette smokers with periodontitis (*n* = 18, 46.3 y.o., ≥10 cigarettes/day for more than 10 years)	Gingival mucosa from periodontal surgical sites and tooth extraction sites	Histomorphometric analysis	No significant changes in vascular density and lumen area when compared with age-matched non-smokers

## Data Availability

No new data were created or analyzed in this study. Data sharing is not applicable to this article.
